# Hydromagnetic Steady Flow of Maxwell Fluid over a Bidirectional Stretching Surface with Prescribed Surface Temperature and Prescribed Surface Heat Flux

**DOI:** 10.1371/journal.pone.0068139

**Published:** 2013-07-12

**Authors:** Sabir Ali Shehzad, Ahmad Alsaedi, Tasawar Hayat

**Affiliations:** 1 Department of Mathematics, Quaid-i-Azam University, Islamabad, Pakistan; 2 Nonlinear Analysis and Applied Mathematics (NAAM) Research Group, Faculty of Science, King Abdulaziz University, Jeddah, Saudi Arabia; National Institute of Genomic Medicine, Mexico

## Abstract

This paper investigates the steady hydromagnetic three-dimensional boundary layer flow of Maxwell fluid over a bidirectional stretching surface. Both cases of prescribed surface temperature (PST) and prescribed surface heat flux (PHF) are considered. Computations are made for the velocities and temperatures. Results are plotted and analyzed for PST and PHF cases. Convergence analysis is presented for the velocities and temperatures. Comparison of PST and PHF cases is given and examined.

## Introduction

Interest of recent researchers in analysis of boundary layer flows over a continuously moving surface with prescribed surface temperature or heat flux has increased substantially during the last few decades. These flows have abundant applications in many metallurgical and industrial processes. Specific examples of such industrial and technological processes include wire-drawing, glass-fiber and paper production, the extrusion of polymer sheets, the cooling of a metallic plate in a cooling bath, drawing of plastic films etc. Such situations occur in the class of flow problems relevant to the polymer extrusion in which the flow is generated by stretching of plastic surface [1], [2]. In addition, internal heat generation/absorption has key role in the heat transfer from a heated sheet in several practical aspects. The heat generation/absorption effects are also important in the flow problems dealing with the dissociating fluids. Influences of heat generation/absorption may change the temperature distribution which corresponds to the particle deposition rate in electronic chips, nuclear reactors, semiconductor wafers etc. The idea of boundary layer flow over a moving surface was introduced by Sakiadis [3]. He discussed the boundary layer flow of viscous fluid over a solid surface. This analysis was extended by Crane [4] for a linearly stretched surface. He provided the closed form solutions of two-dimensional boundary layer flow of viscous fluid over a surface. Numerous literature now exists on the boundary layer flow with heat transfer and in the presence of heat generation/absorption effects (see [5]–[10] and many refs. therein).

A large number of industrial fluids like polymers, soaps, molten plastics, sugar solutions pulps, apple sauce, drilling muds etc. behave as the non-Newtonian fluids [11]. The Navier-Stokes equations cannot explore the properties of such materials. In the literature, different types of fluids models are developed according to the nature of fluids. The non-Newtonian fluids are mainly divided into three categories which are known as the differential, rate and integral types. The fluid considered here is called the Maxwell fluid. It is subclass of rate type fluids predicting the characteristics of relaxation time. The properties of polymeric fluids can be explored by Maxwell model for small relaxation time. Zierep and Fectecau [12] discussed the energetic balance for the Rayleigh-Stokes problem involving Maxwell fluid. Closed form solutions of unsteady flow of Maxwell fluid due to the sudden movement of the plate was described by Hayat et al. [13]. Fetecau et al. [14] provided the exact solutions for the unsteady flow of Maxwell fluid. Here they considered that the flow is generated due to the constantly accelerating plate. Flow of Maxwell fluid with fractional derivative model between two coaxial cylinders was also addressed by Fetecau et al. [15]. Here the inner cylinder is subjected to the time-dependent longitudinal shear stress generating the fluid motion. Helical unidirectional flows of Maxwell fluid due to shear stresses on the boundary have been studied by Jamil and Fetecau [16]. They provided the exact solution by Hankel transform method. Stability analysis for the flow of Maxwell fluid under soret-driven double-diffusive convection in a porous medium was examined by Wang and Tan [17]. Two-dimensional boundary layer flow of Maxwell fluid over a linearly stretching surface was analyzed by Hayat et al. [18]. Mukhopadhyay [19] presented an analysis for the unsteady flow of Maxwell fluid in a porous medium with suction/injection. Falkner-Skan flow of Maxwell fluid with mixed convection over a surface was analytically discussed by Hayat et al. [20].

The main theme of present analysis is to discuss the steady three-dimensional boundary layer flow of Maxwell fluid over a bidirectional stretching surface subject to prescribed surface temperature and prescribed surface heat flux. The effects of applied magnetic field are also included in this analysis. To our knowledge, not much is known about flows induced by a bidirectional stretching surface. Wang [21] discussed the three-dimensional flow of viscous fluid over a bidirectional stretching surface. Ariel [22] provided the exact and homotopy perturbation solution for ref. [21]. Liu and Andersson [23] discussed the heat transfer analysis over a bidirectional stretching surface with variable thermal conditions. Ahmed et al. [24] extended the analysis of ref. [23] for hydromagnetic flow in a porous medium. They presented the series solutions. Hayat et al. and Shehzad et al. [25], [26] studied the boundary layer flows of Maxwell and Jeffery fluids over a bidirectional stretching surface. The present analysis is arranged as follows. The next section contains the mathematical formulation of the problem. Sections three and four are for the homotopy solutions (HAM) [27]–[34], convergence study and discussion. Both cases of prescribed surface temperature (PST) and prescribed surface heat flux (PHF) are given due attention in the discussion section. The main observations of this research are listed in the last section. Further, the correct modelling for magnetohydrodynamic case of Maxwell fluid is given.

## Flow Model

**Figure 1:**
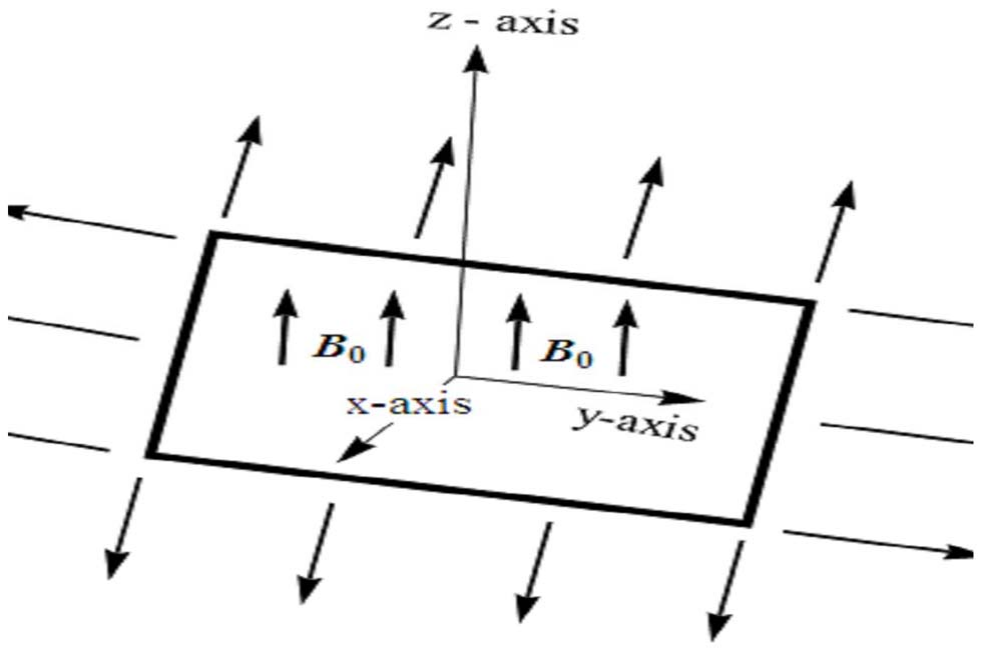
Physical model.



Consider three-dimensional magnetohydrodynamic (MHD) boundary layer flow of an incompressible Maxwell fluid. The flow is induced by bidirectional stretching surface (at 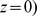 with PST and PHF. Steady flow of an incompressible Maxwell fluid is considered for 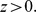 Flow analysis is carried out in the presence of heat generation/absorption parameter. The fluid is electrically conducting in the presence of applied magnetic field with constant strength 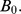 No electric field contribution is taken into account. Induced magnetic field effects are ignored through large magnetic Reynolds number consideration. The geometry of considered flow is shown in Fig. 1. The conservation of mass, momentum and energy for steady flow in presence of magnetic field and heat source/sink can be expressed as

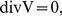
(1)

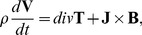
(2)

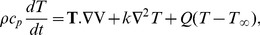
(3)in which 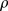 depicts the density, 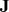 the current density, 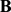 the magnetic field in the 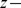 direction, 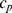 the specific heat, 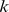 the thermal conductivity and 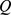 the heat generation/absorption parameter with 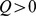 (heat generation) and 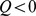 (heat absorption). 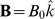 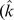 is a unit vector parallel to the 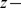 axis). The definition of 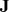 for present flow consideration is
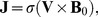
(4)where 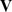 denotes the fluid velocity and 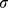 the electrical conductivity. The Lorentz force thus reduces to
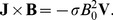
(5)


Expressions of Cauchy 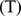 and extra stress 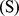 tensors in Maxwell fluid are [11]:
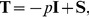
(6)

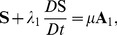
(7)where 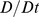 is the Covariant differentiation and 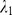 is the relaxation time. The first Rivilin Ericksen tensor 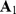 is defined as



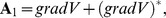
where * indicates the matrix transpose and the velocity field 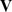 here is taken as



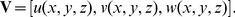
(8)The definition of 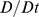 is [11]

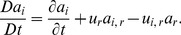
(9)


Following the procedure of ref. [11] at pages 221–223 and using above equations, we have the following scalar expressions
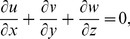
(10)

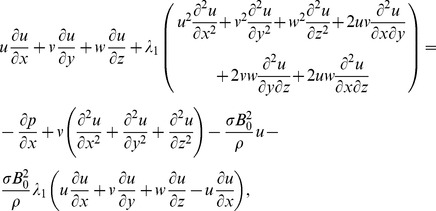
(11)

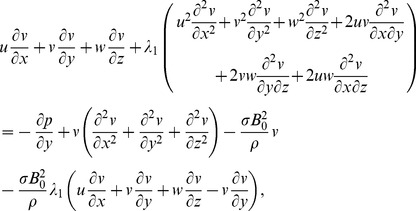
(12)

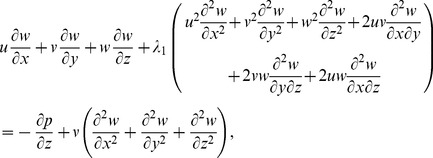
(13)

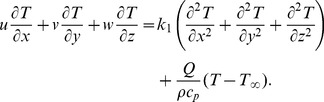
(14)


After employing the boundary layer assumptions [35], the above equations in the absence of pressure gradient yield
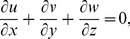
(15)

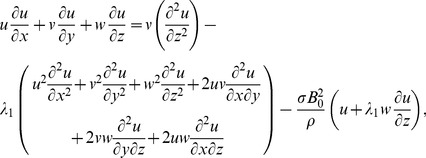
(16)

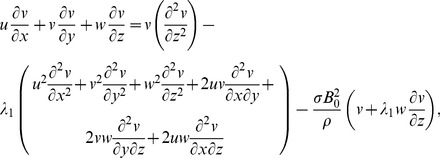
(17)

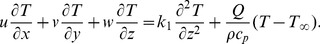
(18)


The associated boundary conditions are defined as follows.
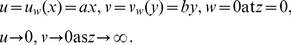
(19)


For temperature, the boundary conditions are specified as [23], [24]:

### 

#### Type i

Prescribed surface temperature (PST)
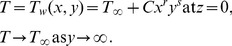
(20)


#### Type ii

Prescribed surface heat flux (PHF)
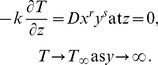
(21)


Here 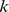 is the thermal conductivity of the fluid, 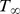 the constant temperature outside the thermal boundary layer, 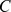 and 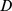 the positive constants. The power indices 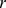 and 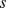 determine how the temperature or the heat flux varies in the 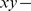 plane.

Following [23], [24] similarity variables for the velocity field are introduced as
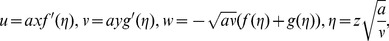
(22)and the temperature similarity variables take different forms depending on the boundary conditions being considered. These are
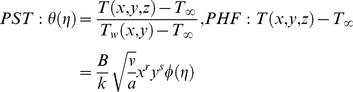
(23)
equation (15) is automatically satisfied and Eqs. (16)–(21) take the following forms:



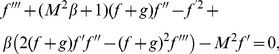
(24)

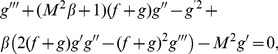
(25)

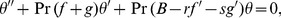
(26)

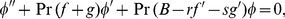
(27)

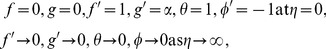
(28)where 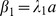 is the Deborah number, 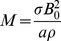 the magnetic parameter, 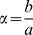 the ratio of stretching rates, 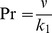 the Prandtl number, 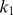 the thermal diffusivity and 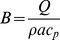 the internal heat parameter.

## Homotopy Analysis Solutions

In this section, we solve the problem consisting of Eqs. (24)–(27) with boundary conditions in Eq. (28) by HAM. For that the initial guesses and auxiliary linear operators are taken as follows:
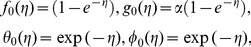
(29)

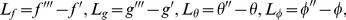
(30)subject to the properties
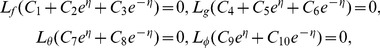
(31)where 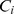 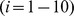 are the arbitrary constants.

At zeroth order, the problems satisfy
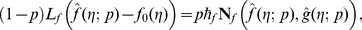
(32)

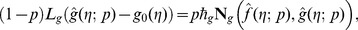
(33)

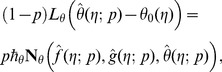
(34)

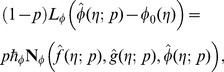
(35)

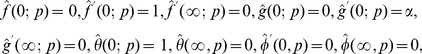
(36)

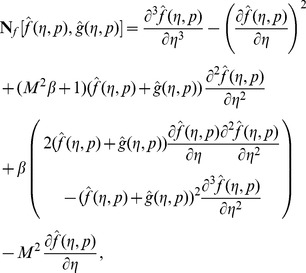
(37)

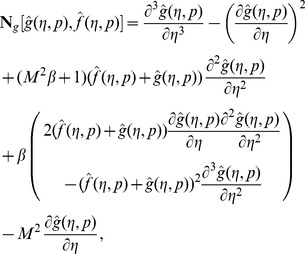
(38)

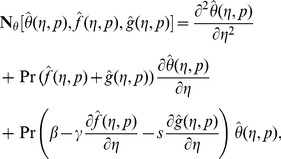
(39)

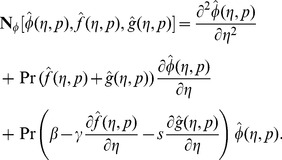
(40)


In above expressions, 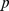 shows the embedding parameter, 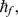 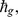 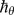 and 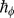 the non-zero auxiliary parameters and 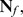 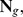 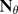 and 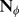 the nonlinear operators. When 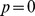 and 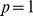 then we obtain
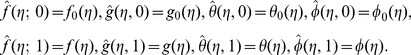
(41)


It should be pointed out that when 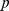 increases from 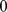 to 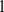 then 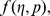 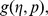 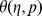 and 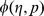 vary from 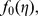 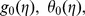 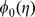 to 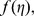 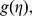 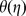 and 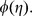 Using Taylors' expansion we write
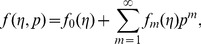
(42)

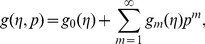
(43)

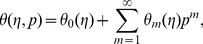
(44)

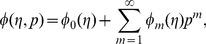
(45)

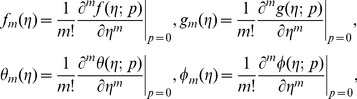
(46)where the parameters 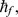 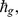 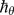 and 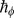 have a key role in the convergence of series solutions. The values of parameters are chosen in such a manner that Eqs. 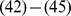 converge at 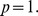 Hence Eqs. 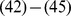 give



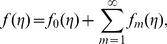
(47)

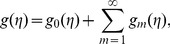
(48)

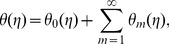
(49)

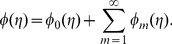
(50)


The general solutions are arranged as follows
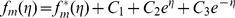
(51)

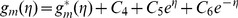
(52)

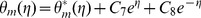
(53)

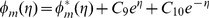
(54)in which the special solutions are denoted by 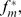 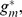 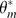 and 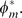


## Convergence of Series Solutions and Discussion

**Figure 2:**
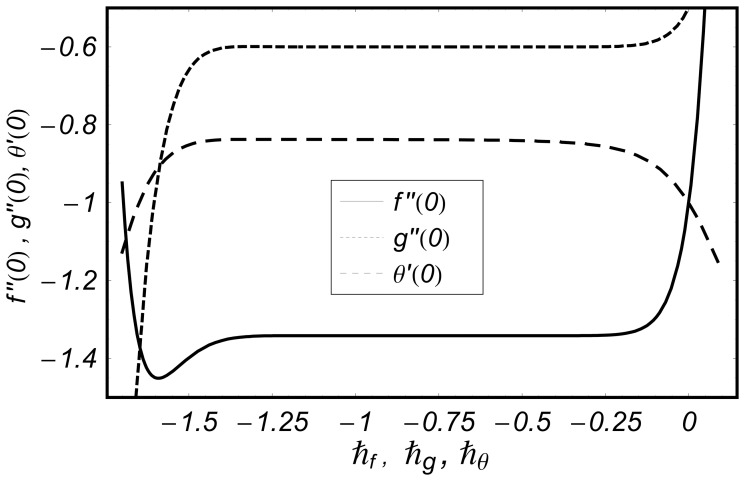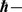 curves for the functions 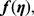 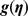 and 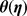 when 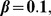 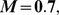 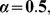 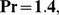 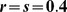 and 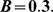

**Figure 3:**
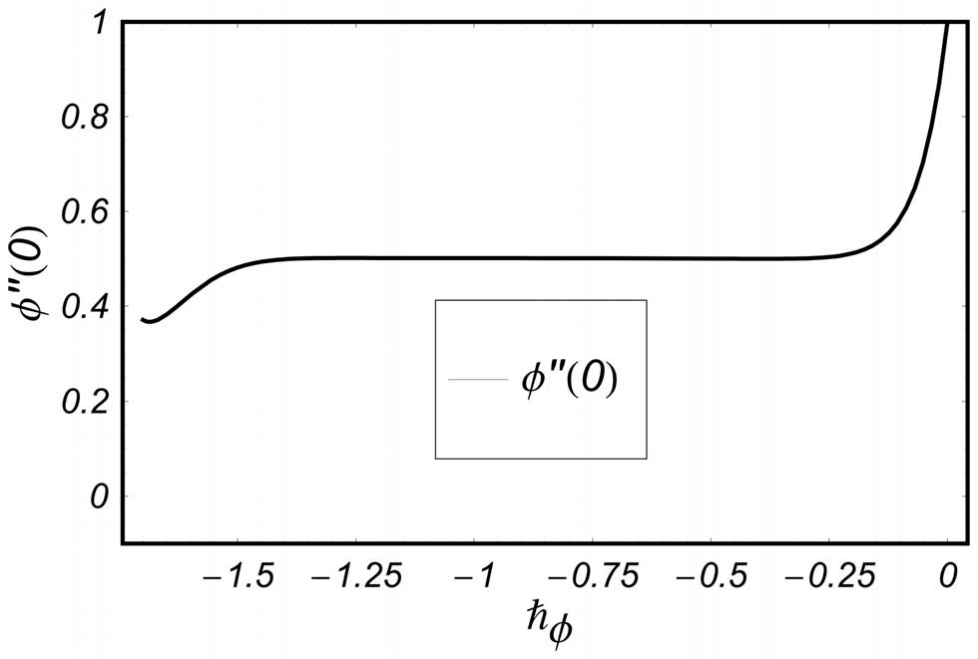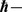 curve for the function 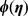 when 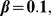 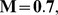 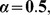 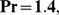 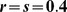 and 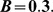

It is well known fact that the homotopy analysis method has a great freedom to choose the auxiliary parameters 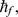 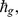 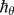 and 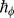 for adjusting and controlling the convergence of series solutions. To determine the appropriate convergence interval of the constructed series solutions, the 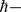 curves at 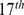 -order of approximations are sketched. Figs. 2 and 3 clearly show that the range of admissible values of 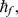 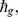 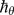 and 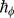 are 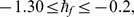 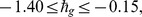 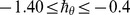 and 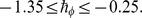


**Figure 4:**
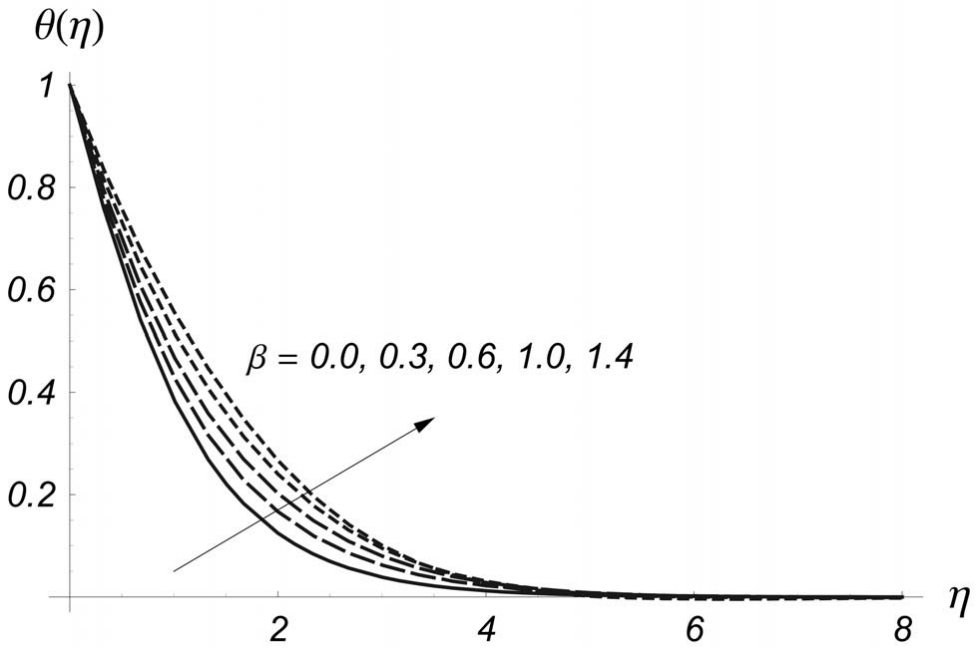
Influence of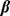 on 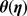 when 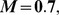 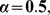 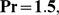 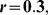 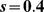 and 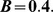

**Figure 5:**
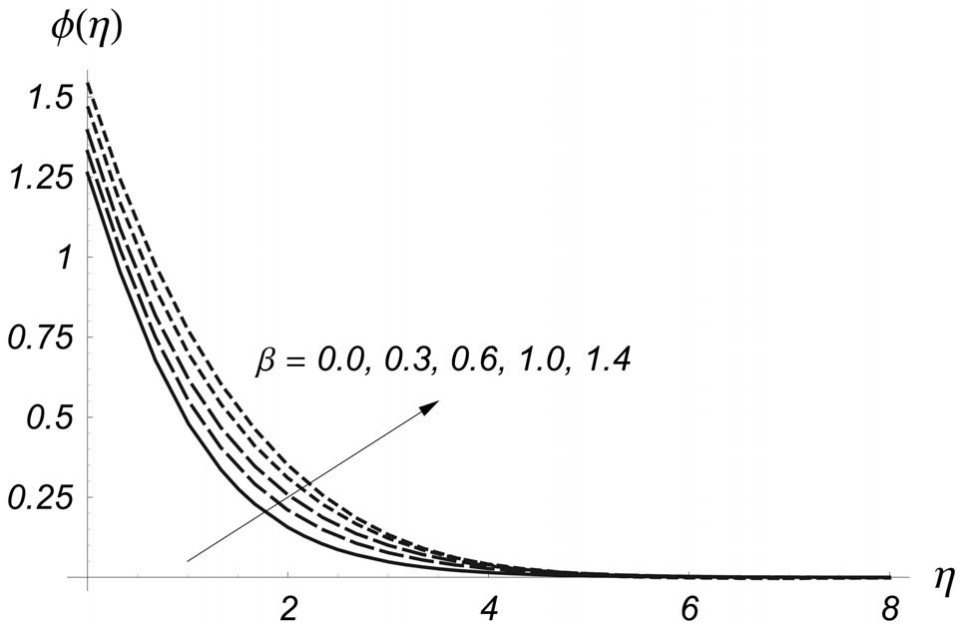
Influence of  on 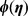 when 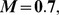 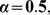 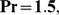 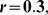 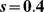 and 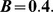

**Figure 6:**
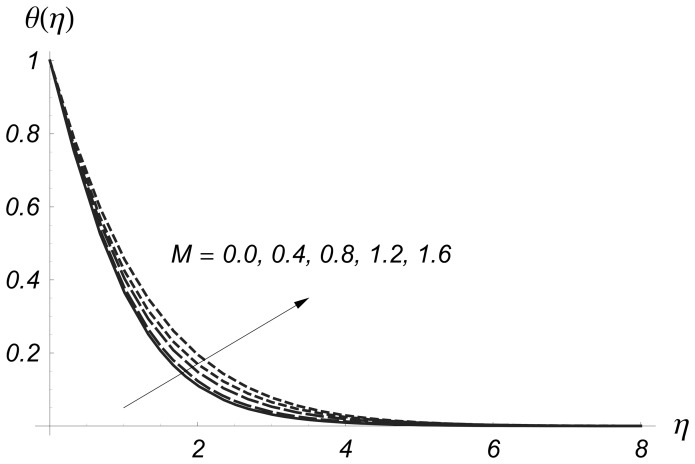
Influence of 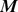 on 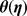 when 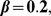 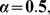 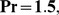 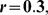 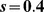 and 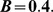

**Figure 7:**
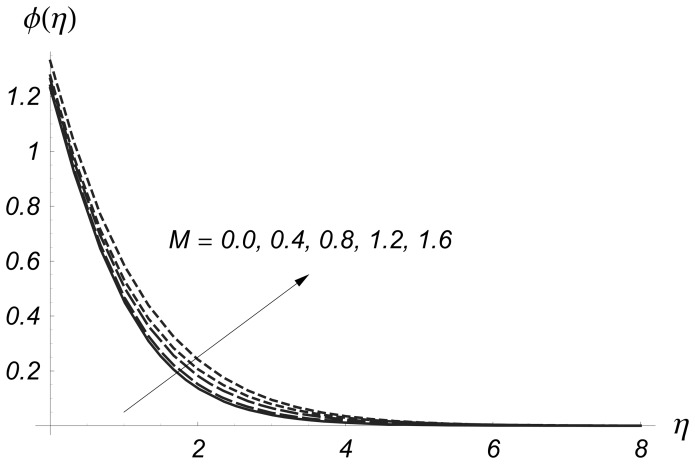
Influence of 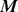 on 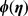 when 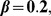 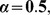 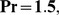 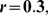 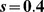 and 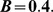

**Figure 8:**
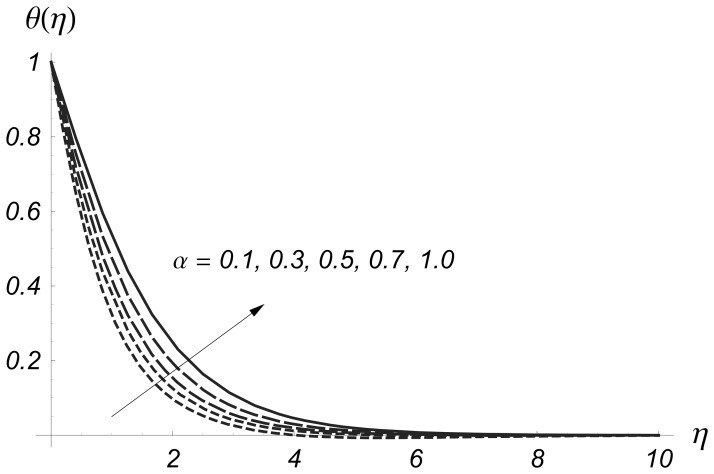
Influence of 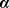 on 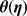 when 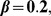 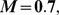 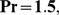 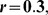 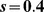 and 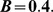

**Figure 9:**
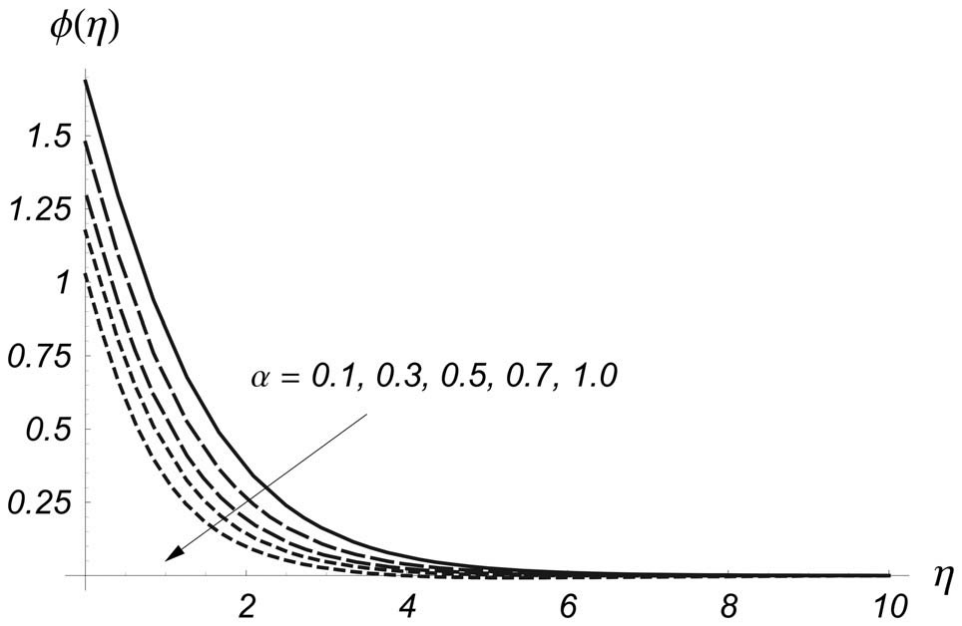
Influence of 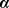 on 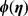 when 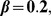 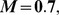 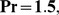  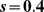 and 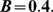

**Figure 10:**
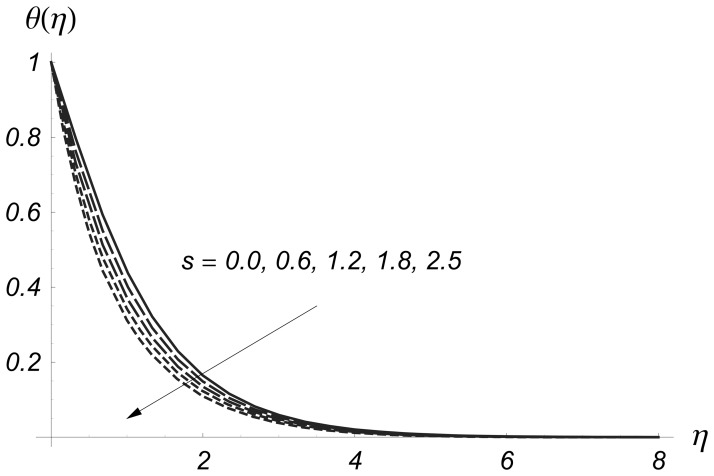
Influence of 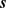 on 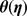 when 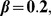 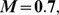 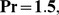 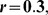 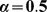 and 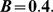

**Figure 11:**
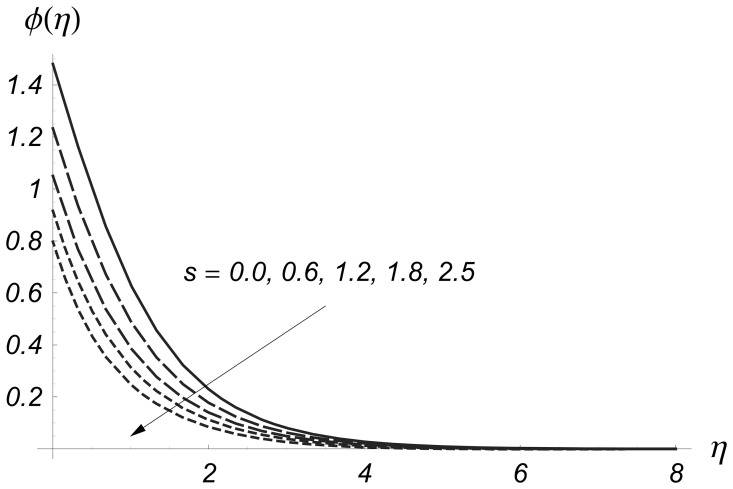
Influence of 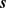 on 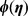 when 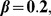 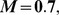 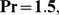 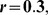 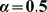 and 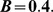

**Figure 12:**
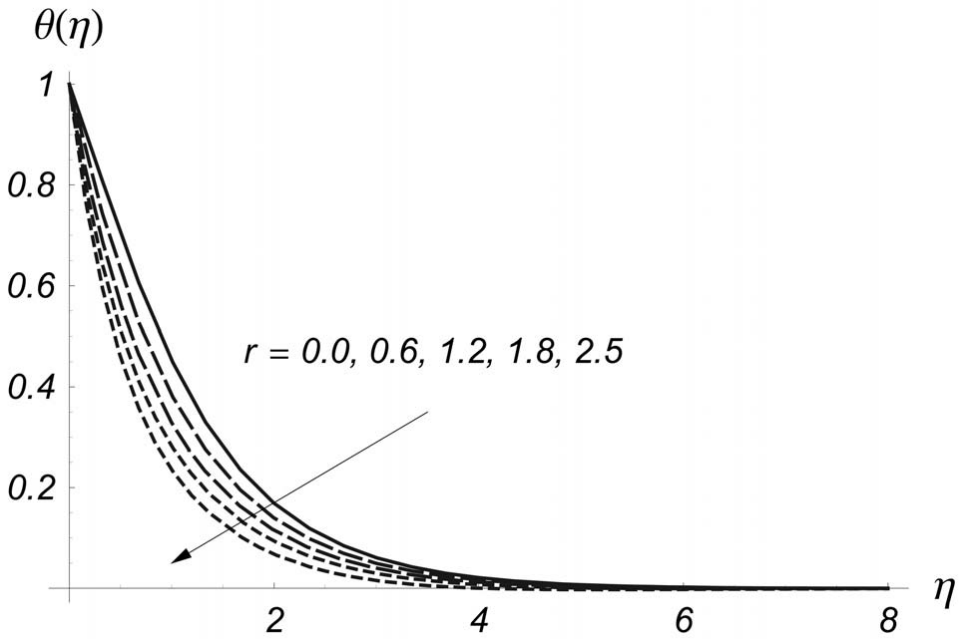
Influence of 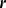 on 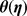 when 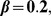 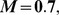 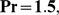 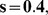 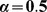 and 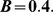

**Figure 13:**
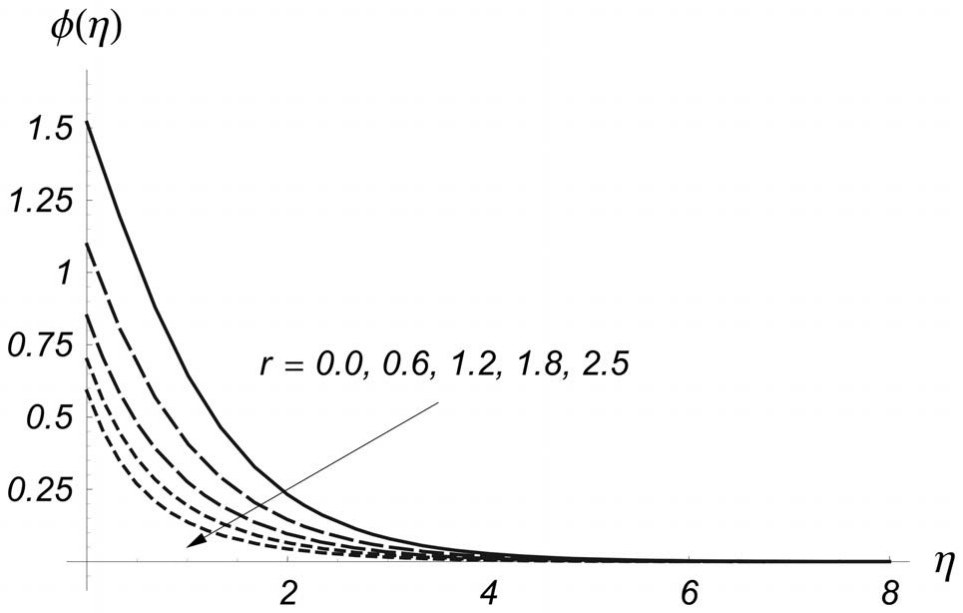
Influence of 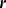 on 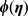 when 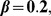 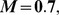 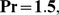 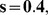 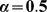 and 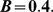

**Figure 14:**
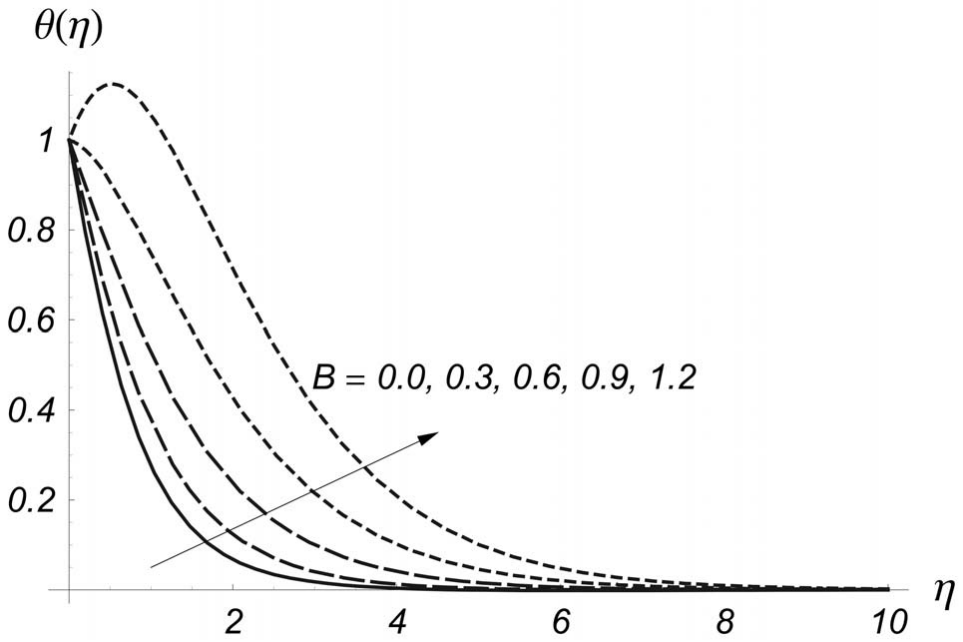
Influence of 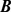 on 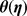 when 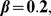 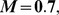 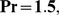 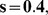 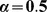 and 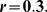

**Figure 15:**
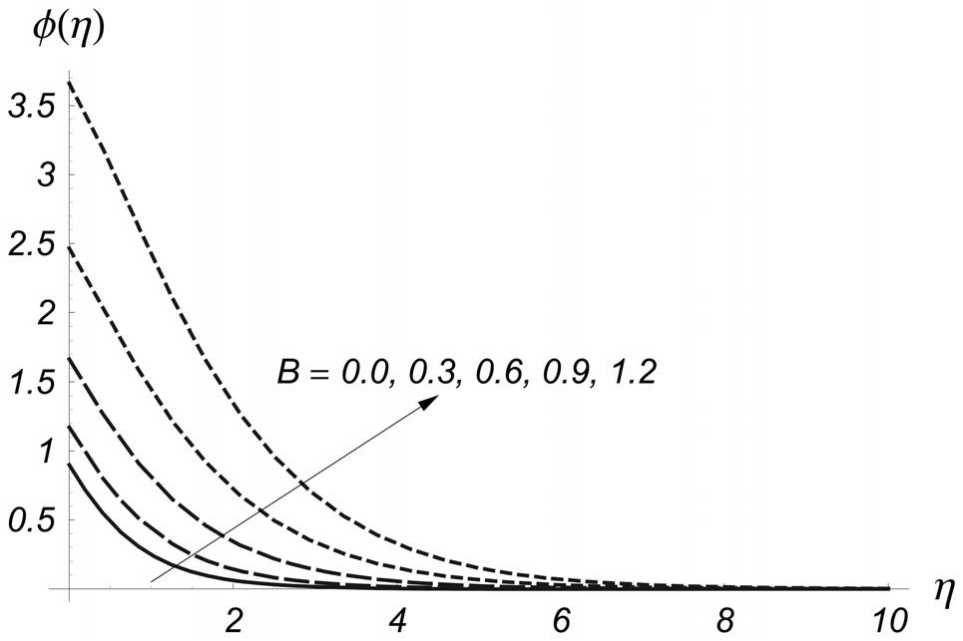
Influence of 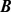 on 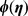 when 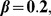 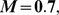 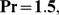 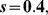 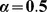 and 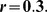

**Figure 16:**
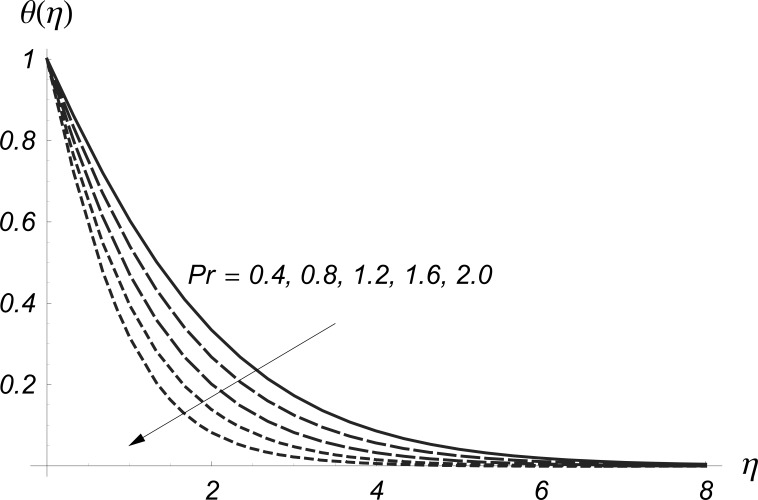
Influence of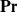 on 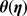 when 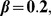 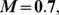 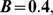 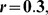 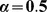 and 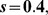

**Figure 17:**
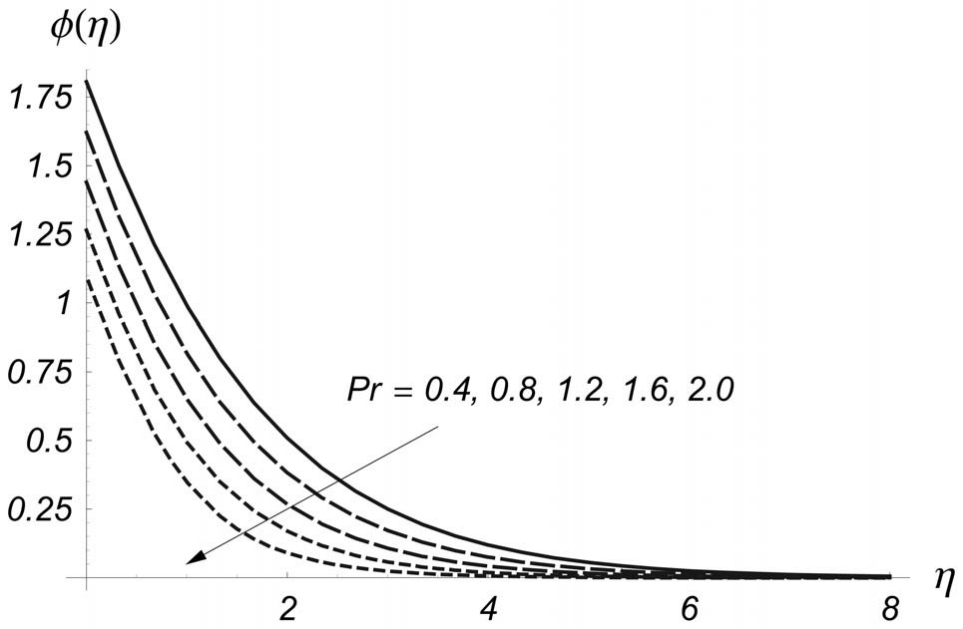
Influence of 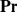 on 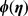 when 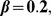 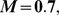 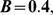 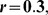 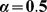 and 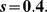

The results are displayed graphically to see the effects of 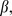 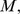 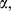 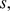 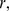 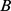 and 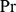 on the prescribed surface temperature and prescribed surface heat flux. We denote temperature variation for PST case by 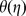 and for PHF situation by 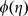 in the Figs. 4–17. Figs. 4 and 5 illustrate the variations of Deborah number on 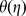 and 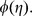 From these Figs., we have seen that both 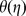 and 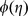 are increased with an increase in 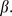 Deborah number is based on the relaxation time. When Deborah number increases, the relaxation time increases. This increase in relaxation time causes an increase in 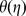 and 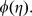 Comparison of Figs. 4 and 5 shows that 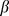 has similar effects on 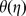 and 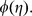 Figs. 6 and 7 are plotted to see the effects of magnetic parameter 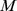 on 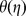 and 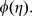 Clearly the thermal boundary layer thicknesses are increased for larger values of magnetic parameter. In fact the magnetic parameter involves the Lorentz force. Larger values of magnetic parameter correspond to the stronger Lorentz force. This stronger Lorentz force give rise to the thermal boundary layer thicknesses. Figs. 8 and 9 illustrate the variations of 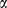 on 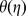 and 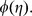 From these Figs. it is noticed that both 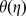 and 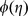 are reduced when we increased the values of 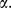 Also the thermal boundary layer becomes thinner for higher values of 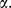 This reduction in thermal boundary layer for larger values of 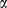 is due to the entertainment of cooler to ambient fluid. The power indices 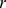 and 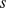 control the non-uniformity of the surface temperature in the prescribed surface temperature situation. Figs. 10 and 11 depict that 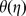 and 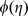 are decreasing functions of 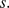 Also we noted that 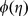 reduces rapidly as comparison to 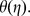 Effect of 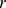 on 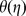 and 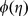 are seen in the Figs. 12 and 13. The values of 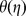 and 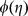 are reduced when values of 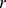 are increased. It is concluded that the non-uniformity of the sheet temperature has prominent effect on the temperature fields for the reduction in temperature and thinner thermal boundary layer. Comparison of Figs. 12 and 13 illustrates that the variations in 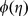 are more pronounced when compared to the variations in 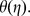 Also we examined that 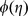 at the wall reduced rapidly when the values of 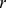 are larger. Figs. 14 and 15 depict the variations of heat generation/absorption parameter 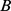 on 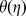 and 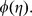 Both 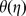 and 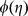 are increased by increasing values of heat generation/absorption parameter. Physically an increase in heat generation/absorption parameter produced more heat due to which the temperature of fluid increases. This increase in temperature gives rise to 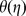 and 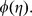 The effects of Prandtl number on 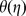 and 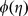 are analyzed in the Figs. 16 and 17. These Figs. clearly show that 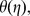 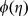 and their related thermal boundary layer thicknesses are reduced for the larger values of Prandtl number 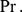 Obviously the Prandtl number depends upon the thermal diffusivity. Larger values of Prandtl number give smaller thermal diffusivity and consequently the values of 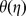 and 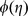 decrease.

Table 1 has been prepared to analyze the convergent values of the velocities, 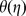 and 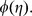 We have seen that our solutions for velocities converge from 16th order of approximations whereas one needs 25th order of deformations for 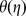 and 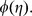 Hence we need less deformations for the velocities in comparison to temperatures for a convergent solution. Table 2 provides the values of temperature gradient 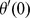 for different values of 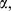 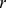 and 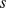 when 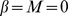 and 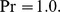 One can see that our solutions has an excellent agreement with the previous results in a limiting case [20], [21]. Further, it is observed that the temperature gradient at surface 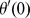 becomes positive and reduces for 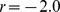 and 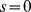 and negative for 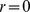 and 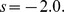 Table 3 presents the numerical values of 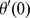 and 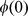 for different values of 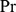 and 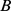 when 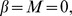 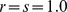 and 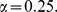 From this Table we noted that our series solutions have very good agreement with the previous results available in the literature.

**Table 1:** Convergence analysis of series solutions by numerical data for different order of deformations when 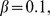 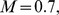 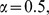 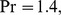 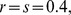 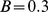 and 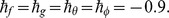

Order of deformations	f′′(0)	g′′(0)	θ′(0)	φ′(0)
1	−1.345900	−0.592325	−0.92800	0.55000
10	−1.341759	−0.600119	−0.84012	0.50038
16	−1.341761	−0.600122	−0.83823	0.50111
25	−1.341761	−0.600122	−0.83775	0.50128
30	−1.341761	−0.600122	−0.83775	0.50128
35	−1.341761	−0.600122	−0.83775	0.50128
40	−1.341761	−0.600122	−0.83775	0.50128

**Table 2:** Temperature gradient at surface 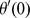 for different values of 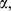 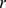
and 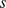 with 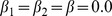 and 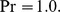

		r = s = 0	r = −2, s = 0	r = 2, s = 0	r = 0, s = −2	r = 0, s = 2
[23]	α = 0.25	−0.665933	0.554512	−1.364890	−0.413111	−0.883125
[24]		−0.665927	0.554573	−1.364890	−0.413101	−0.883123
Present		−0.66593	0.55457	−1.36489	−0.41310	−0.88312
[23]	α = 0.50	−0.735334	0.308578	−1.395356	−0.263381	−1.106491
[24]		−0.735333	0.308590	−1.395357	−0.263376	−1.106500
Present		−0.73533	0.30858	−1.39536	−0.26338	−1.10649
[23]	α = 0.75	−0.796472	0.135471	−1.425038	−0.126679	−1.292003
[24]		−0.796470	0.135470	−1.425037	−0.126679	−1.292010
Present		−0.79472	0.13547	−1.42504	−0.12667	−1.29200

**Table 3:** Temperature gradient at surface 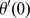 and 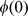 for different values of 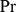 and 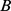 when 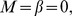 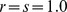 and 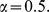

		−θ′(0) for PST			φ(0) for PHF		
		B = −0.2	B = 0.0	B = 0.2	B = −0.2	B = 0.0	B = 0.2
[23]	Pr = 1.0	1.348064	1.255781	1.148932	0.741805	0.796317	0.870355
[24]		1.348064	1.255780	1.148934	0.741808	0.796318	0.870372
Present		1.34806	1.25578	1.14893	0.74180	0.79632	0.87037
[23]	Pr = 5.0	3.330392	3.170979	3.002380	0.300265	0.315360	0.333069
[24]		3.330394	3.170981	3.002384	0.300265	0.315363	0.333071
Present		3.33039	3.17098	3.00238	0.30028	0.31537	0.33308
[23]	Pr = 10.0	4.812149	4.597141	4.371512	0.207807	0.217527	0.228754
[24]		4.812151	4.597143	4.371516	0.207809	0.217529	0.228756
Present		4.81215	4.59714	4.37152	0.20781	0.21753	0.22876

## Concluding Remarks

In this study, the three-dimensional MHD flow of Maxwell fluid generated by bidirectional stretching surface is investigated for two cases of prescribed surface temperature (PST) and prescribed surface heat flux (PHF). The effects of applied magnetic field 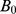 are also taken into account. Interesting observations of this study can be mentioned below:

- Effects of Deborah number 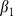 on 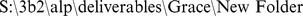 and 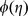 are similar in a qualitative manner.
- Both 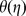 and 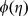 are increasing functions of magnetic parameter 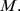

- Increase in ratio parameter 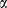 reduces the temperatures and their boundary layer thicknesses.
- Temperature for 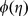 case decreases rapidly in comparison to 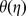 case when larger values of 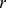 and 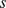 are employed.
- An increase in heat generation/absorption parameter enhances the temperatures 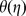 and 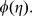

- Our series solutions have an excellent agreement with the previous results in limiting cases.

## References

[1] Fisher EG (1976) Extrusion of plastics. Wiley, New York.

[2] Tadmor Z, Klein I (1970) Engineering priciples of plasticating extrusion, in polymer science and engineering series. Van Nastrand Reinhold, New York.

[3] Sakiadis BC (1961) Boundary layer behavior on continuous solid surfaces: I Boundary layer equations for two dimensional and axisymmetric flow. AIChE J 7: 26–28.

[4] Crane LJ (1970) Flow past a stretching plate, ZAMP. 21: 645–647.

[5] Kazem S, Shaban M, Abbasbandy S (2011) Improved analytical solutions to a stagnation-point flow past a porous stretching sheet with heat generation. J Franklin Institute 348: 2044–2058.

[6] Mukhopadhyay S, Bhattacharyya K, Layek GC (2011) Slip effects on boundary layer stagnation point flow and heat transfer towards a shrinking sheet. Int J Heat Mass Transfer 54: 2751–2757.

[7] Rashidi MM, Pour SAM, Abbasbandy S (2011) Analytic approximate solutions for heat transfer of a micropolar fluid through a porous medium with radiation. Commun Nonlinear Sci Numer Simulat 16 (2011) 1874–1889.

[8] Hayat T, Shehzad SA, Qasim M, Obaidat S (2012) Radiative flow of a Jeffery fluid in a porous medium with power law heat flux and heat source. Nuclear Eng Design 243: 15–19.

[9] Turkyilmazoglu M (2011) Thermal radiation effects on the time-dependent MHD permeable flow having variable viscosity. Int J Thermal Sci 50: 88–96.

[10] Makinde OD, Aziz A (2011) Boundary layer flow of a nonofluid past a stretching sheet with a convective boundary condition. Int J Thermal Sci 50: 1326–1332.

[11] Harris J (1977) Rheology and non-Newtonian flow. Longman, London.

[12] Zierep J, Fetecau C (2007) Energetic balance for the Rayleigh – Stokes problem of a Maxwell fluid. Int J Eng Sci 45: 617–627.

[13] Hayat T, Fetecau C, Abbas Z, Ali N (2008) Flow of a Maxwell fluid between two side walls due to a suddenly moved plate. Nonlinear Analysis: Real World Applications 9: 2288–2295.

[14] Fetecau C, Athar M, Fetecau C (2009) Unsteady flow of a generalized Maxwell fluid with fractional derivative due to a constantly accelerating plate. Comput Math Appl 57: 596–603.

[15] Fetecau C, Fetecau C, Jamil M, Mahmood A (2011) Flow of fractional Maxwell fluid between coaxial cylinders. Arch Appl Mech 81: 1153–1163.

[16] Jamil M, Fetecau C (2010) Helical flows of Maxwell fluid between coaxial cylinders with given shear stresses on the boundary. Nonlinear Analysis: Real World Applications 11: 4302–4311.

[17] Wang S, Tan WC (2011) Stability analysis of Soret-driven double-diffusive convection of Maxwell fluid in a porous medium. Int J Heat Fluid Flow 32: 88–94.

[18] Hayat T, Shehzad SA, Qasim M, Obaidat S (2011) Steady flow of Maxwell fluid with convective boundary conditions. Z Naturforsch A 66a: 417–422.

[19] Mukhopadhyay S (2012) Upper-convected Maxwell fluid flow over an unsteady stretching surface embedded in porous medium subjected to suction/blowing. Z Naturforsch A 67a: 641–646.

[20] Hayat T, Farooq M, Iqbal Z, Alsaedi A (2012) Mixed convection Falkner-Skan flow of a Maxwell fluid. J Heat Transfer-Trans ASME 134: 114504.

[21] Wang CY (1984) The three-dimensional flow due to a stretching sheet. Phys Fluids 27: 1915–1917.

[22] Ariel PD (2007) The three-dimensional flow past a stretching sheet and the homotopy perturbation method. Comput Math Appl 54: 920–925.

[23] Liu IC, Andersson HI (2008) Heat transfer over a bidirectional stretching sheet with variable thermal conditions. Int J Heat Mass Transfer 51: 4018–4024.

[24] Ahmad I, Ahmed M, Abbas Z, Sajid M (2011) Hydromagnetic flow and heat transfer over a bidirectional stretching surface in a porous medium. Thermal Sci 15: S205–S220.

[25] Hayat T, Shehzad SA, Alsaedi A (2012) Study on three dimensional flow of Maxwell fluid over a stretching sheet with convective boundary conditions. Int J Physical Sci 7: 761–768.

[26] Shehzad SA, Alsaedi A, Hayat T (2012) Three-dimensional flow of Jeffery fluid with convective surface boundary conditions. Int J Heat Mass Transfer 55: 3971–3976.

[27] Liao SJ (2009) Notes on the homotopy analysis method: Some definitions and theorems. Commun Nonlinear Sci Numer Simulat 14: 983–997.

[28] Turkyilmazoglu M (2012) Solution of the Thomas-Fermi equation with a convergent approach. Commun Nonlinear Sci Numer Simulat 17: 4097–4103.

[29] Turkyilmazoglu M (2012) The Airy equation and its alternative analytic solution. Phys Scr 86: 055004.

[30] Turkyilmazoglu M (2011) Convergence of the homotopy perturbation method. Int J Nonlinear Sci Numer Simulat 12: 9–14.

[31] Turkyilmazoglu M (2011) Numerical and analytical solutions for the flow and heat transfer near the equator of an MHD boundary layer over a porous rotating sphere. Int J Thermal Sci 50: 831–842.

[32] Rashidi MM, Keimanesh M, Rajvanshi SC (2012) Study of pulsatile flow in a porous annulus with the homotopy analysis method. Int J Numer Methods Heat Fluid Flow 22: 971–989.

[33] Hayat T, Shehzad SA, Alsaedi A, Alhothuali MS (2012) Mixed convection stagnation point flow of Casson fluid with convective boundary conditions. Chin Phys Lett 29: 114704.

[34] Hayat T, Shehzad SA, Alsaedi A (2012) Soret and Dufour effects on magnetohydrodynamic (MHD) flow of Casson fluid. Appl Math Mech-Engl Edit 33: 1301–1312.

[35] Schichting H (1964) Boundary layer theory. 6th edition, McGraw-Hill, New York.

